# Does the repeat dose of gonadotropin-releasing hormone agonist trigger in polycystic ovarian syndrome improve in vitro fertilization cycles outcome? A clinical trial study

**DOI:** 10.18502/ijrm.v13i7.7363

**Published:** 2020-07-22

**Authors:** Abbas Aflatoonian, Fatemeh Haghighi, Masrooreh Hoseini, Saeid Haghdani

**Affiliations:** ^1^Department of Obstetrics and Gynecology, Research and Clinical Center for Infertility, Yazd Reproductive Sciences Institute, Shahid Sadoughi University of Medical Science, Yazd, Iran.; ^2^Department of Urology, Hasheminejad Kidney Research Center (HKRC), Iran University of Medical Science, Tehran, Iran.

**Keywords:** Polycystic ovarian syndrome, Treatment, In vitro fertilization, Gonadotropin-Releasing hormone.

## Abstract

**Background:**

A repeat dose of Gonadotropin-releasing Hormone (GnRH) agonist could provide long duration of luteinizing hormone (LH) surge and amplitude appropriately.

**Objective:**

Improvement in oocyte maturity could be obtained by a repeat dose of GnRH agonist.

**Materials and Methods:**

In this randomized double-blinded study, 120 women with polycystic ovarian syndrome and serum estradiol level (E2) > 3000 who were candidate for in vitro fertilization with Antagonist protocol were enrolled between July 2018 and July 2019. Participants were randomized in two groups - and final oocyte maturation was triggered with two doses: In group A, a repeat dose of 0.1 mg, 12 hr. after the first dose and in group B, 0.2 mg SC triptorelin (decapeptyl) 35 hr. prior to oocyte retrieval. Serum Estradiol, LH, and progesterone concentration were measured on the trigger day. Serum LH measurement was done three times in both groups. The outcomes were oocyte yield, meiosis (M) I, MII, Maturity rate, germinal vesicle (GV) rate, 2 pronuclear, embryo yield, ovarian hyper stimulation syndrome rates.

**Results:**

Maturity rate (p = 0.89), MI (p = 0.38), MII (p = 0.89), and GV oocytes (p = 0.38) were not statistically different between the two study groups. LH levels measured at 12 hr post-trigger did not relate statistically significant with maturity rate in our participants (p = 0.96). No empty follicular syndrome was reported.

**Conclusion:**

Although, the second dose of GnRH agonist after 12 hr since the first dose could provide duration of LH surge and amplitude and as a result no empty follicular syndrome was seen, the maturity rate, MI, MII, and GV oocytes were not different between the two study groups.

## 1. Introduction 

Gonadotropin-releasing hormone agonists (GnRHa) were introduced in 1990s for final oocyte maturation in in vitro fertilization (IVF) cycles as an alternative to standard human chorionic gonadotropin (HCG) in GnRH antagonist protocol (1, 2). GnRHa trigger is the cause of FSH surge that moves up nuclear maturation (3, 4). HCG with a big carbohydrate content has a luteinizing hormone (LH) like activity with long half-life and persistent LH receptor activity (5); in contrast with HCG, GnRH agonist induces surge of both LH and FSH and has shorter activity that result in notable decrease in the risk of ovarian hyperstimulation syndrome (OHSS) (6, 7). LH surge in natural cycle continues for 48 hr and is divided into three phases: quickly ascension phase, plateau phase that continues for 14 hr, and decreasing phase of 20 hr (8). The LH surge result from the GnRHa trigger included in two phases: small ascending arm (4 hr) and prolonged decreasing arm (20 hr); therefore, decreasing OHSS syndrome attributed to inadequate LH surge (9). On the other hand, empty follicular syndrome (EFS) and immature oocyte syndrome cases were also reported after the GnRHa prescription, which questions the single dose GnRHa efficacy (10, 11).

In addition, in PCOS women and hyper responders after GnRHa trigger, the gonadotropin answer is greatly shorter than endogenic LH surge in a natural cycle that causes more suboptimal expected answer (12, 13). The underlying mechanisms proposed are that some women with PCOS show neuro-endocrine abnormalities; moreover, the supraphysiologic estradiol (E2) level cause apparent increase in the number of follicles and insufficient number of LH receptor (14), therefore, a single dose of GnRHa might not lead to LH surge over a threshold level in these participants. In order to gain optimum responses, repeated dose 12 hr after starting doses of GnRHa resulted in terms of better maturity of oocytes, higher number of blastocysts, and higher clinical pregnancy in Deepikaand colleagues study. In a similar research, we aimed to evaluate if a second dose 12 hr. after the first dose of GnRH agonist trigger could provide long duration of LH surge and amplitude appropriately making an improvement in oocyte maturity.

The aim of this study was to see weather oocyte maturity will improve by a repeat dose of GnRHa or not?

## 2. Materials and Methods

This study is a prospective, randomized double-blind study conducted at Research and Clinical Center for Infertility. All women identified with PCOS were enrolled in this study and IVF was done for them in antagonist protocol between July 2018 and July 2019.

The inclusion criteria were 1) PCOS women outlined based on ESHRE ASRM Rotterdam criteria, 2) stimulation in a GnRH ant protocol, 3) age 20-38 yr, 4) E2 concentration > 3000 IU on trigger day, 5) Body Mass Index > 18 and < 30, 6) indication for IVF, and 7) participants' desire

The exclusion criteria were 1) severe male factor, 2) uterine abnormally, 3) endometriosis, and 4) metabolic disease (ex. Diabetes).

### Study protocol

In PCOS women, controlled ovarian stimulation was started with recombinant FSH (Gonal-f, Merck Serono, Germany) at the dosage of 150 IU/day, beginning day 2 of the cycle. GnRH antagonist-Ganirelix (Orgalotran) 0.25 mg/day subcutaneous (sc) was started in flexible protocol when at least one follicle was > 14 mm. Gonadotropin and antagonist were continued until the trigger day. Follicular development was probed by transvaginal sonography.

Serum E2, LH, and progesterone concentration were measured on the trigger day when three lead follicles reached 17 mm in diameter. Participants with serum E2 > 3000 based on randomization table number were divided in two groups. Final oocyte maturation was triggered with a single dose of 0.2 mg sc triptorelin (decapeptyl) 34-36 hr. prior to oocyte retrieval in both groups. In group A, a repeat dose of 0.1 mg, 12 hr after the first dose was prescribed, but in group B, a single dose of GnRHa 0.2 mg sc triptorelin (decapeptyl) was given. In group A, serum LH was measured 12 hr. after the first dose of GnRHa administration, and then again after the second dose of GnRHa and prior to oocyte retrieval. In group B also, serum LH measurement was measured three times as group A. Transvaginal ultrasound guided oocyte pickup (OPU) was done 35 hr after the first dose agonist. Oocyte retrieval was done under general anesthesia with single lumen oocyte retrieval needle (Swemed, Vitrolife, Sweden). OHSS symptom assessment and transvaginal sonography was done on days 4 and 7 post pick-up to evaluate the ovarian size, free fluid in Douglas. IVF or intracytoplasmic sperm injection was done based on sperm parameter. Fertilization was assessed 18 hr. following IVF with the presence of 2 pronuclei (2PN).

The outcomes were oocyte yield, MI rate, MII rate, maturity rate, germinal vesicle (GV) rate, 2PN, and embryo yield OHSS rates and type were also assessed. Maturity rate was defined as the ratio of MII oocytes (presence of a polar body) to the total number of oocytes. Serum LH (IU/L), E2, and Progesterone (ng/mL) levels were compared between the two groups. All good-quality embryos were frozen.

### Ethical consideration

Signed informed consent was obtained from all participants before enrollment. The study protocol was approved by Shahid Sadoughi University of Medical Science ethical committee (code: IR.SSU.RSI.1397.022).

### Statistical analysis

Data were analyzed with the Statistical Package for the Social Sciences, version 20.0 (SPSS, USA). Independent sample *t* test was used for continuous variables that were normally distributed and Mann-Whitney U-test for non-normally distributed data. The significant level of p-value was considered P < 0.05.

## 3. Results

**Figure 1:**
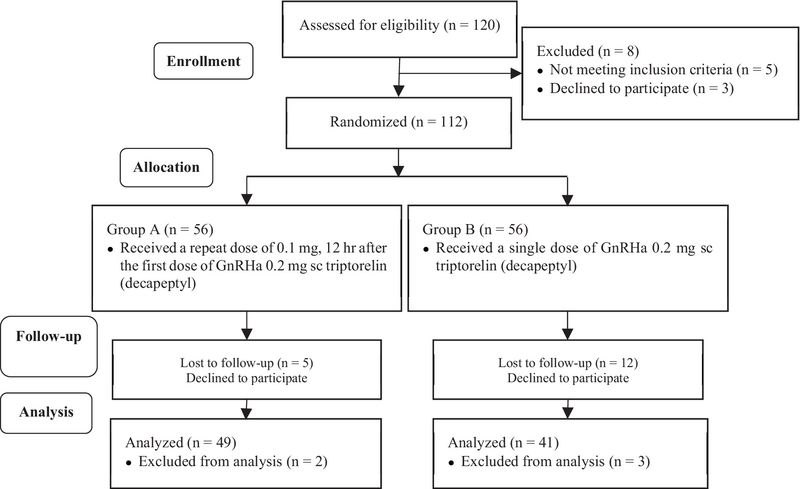
Consort flow diagram.

120 participants were enrolled in the study; 30 were excluded because of the exclusion criteria and missing follow-up. The patients characteristics in both groups A and B are presented in Table I. The consort flow diagram is presented as Figure 1. The percentage of mild and moderate types of OHSS were 23.5% and 76.5%, respectively, in group A, and mild, moderate, and severe OHSS were 29.6%, 66.7%, and 3.7%, respectively, in group B. The outcomes are presented in Table II. As presented in Table II, no variable was statistically different between the two groups.

**Table 1:** Comparison of patients characteristics between group A and group B


**Variables **	**Group A (n = 49) **	**Group B (n = 41)**	**p-value**
**Age**	29.30 ± 4.15	28.95 ± 5.23	0.72*
**BMI**	22.29 ± 4.03	22.27 ± 4.14	0.98*
**Duration of infertility**	6.32 ± 3.11	5.42 ± 3.18	1.8*
**Primary infertility N (%)**	43 (87.8)	35 (85.4)	
**Secondary infertility**	6 (12.2)	6 (14.6)	0.74*
**OHSS **	34 (69.4)	27 (65.9)		0.72*
**AMH**	8.68 ± 3.92	7.48 ± 3.81	0.14*
**Estradiol**	6543.22 ± 588.76	4912.16 ± 333.41	0.04**
**Progesterone**	1.17 ± 0.15	1.11 ± 0.17	0.86**
**LH1**	2.08 ± 0.22	3.02 ± 0.36	0.03**
Data presented as Mean±SD BMI: Body mass index; OHSS: Ovarian hyper stimulation syndrome; AMH: Anti Mulerian hormone; LH: Luteinizing hormone; Independent sample *t* test*, Mann-Whitney U-test**			

**Table 2:** Comparison of outcomes between group A and group B


**Variables **	**Group A (n = 49)**	**Group B (n = 41)**	**p-value**
**Maturity rate**	0.80 ± 0.21	0.79 ± 0.18	0.89*
**Oocyte number**			
**MI**	1.28 ± 0.28	1.62 ± 0.54	0.38**
**MII**	19.83 ± 1.17	15.02 ± 1.10	0.06**
**GV**	3.02 ± 0.55	1.72 ± 0.27	0.38**
**ART method**			
**IVF**	6.89 ± 9.37	5.00 ± 6.66	0.28*
**ICSI**	12.85 ± 7.20	10.41 ± 5.63	0.08*
**2PN**	10.93 ± 7.29	9.46 ± 6.60	0.32*
**Embryo**	9.95 ± 7.23	8.51 ± 5.48	0.29*
**LH2**	50.29 ± 29.19	50.60 ± 35.21	0.96*
**LH3**	15.75 ± 9.77	15.60 ± 11.25	0.94*
**LH4**	4.71 ± 0.43	6.10 ± 1.11	0.66**
Data presented as Mean±SD MI: Meiosis I; MII: Meiosis II; GV: Germinal vesicle; IVF: In vitro fertilization; ICSI: Intra cytoplasmic sperm injection; 2PN 2 pronuclear; LH: Luteinizing hormone; Independent sample *t* test*, Mann-Whitney U-test**			

## 4. Discussion

Oure results showed that the maturity rate, MI, MII, and GV oocytes were not statistically different between the two study groups. LH levels measured at 12 hr. post-trigger did not relate statistically significant with maturity rate in our participants also no empty follicular syndrome was reported. It is identified that the restart of meiosis at 18 hr. after the onset of LH surge (15), and for the highest oocyte maturation, LH concentration should be retained over a threshold for 14-27 hr. A shorter duration and low-level LH concentration post-trigger with single dose of GnRHa cannot maintain an LH level over threshold for 14-27 hr, thus insufficient to induce best oocyte maturation. This suboptimal response can be more probable in women with PCOS, a condition with hypothalamic-pituitary-ovarian axis dysfunction. Deepika and colleagues claimed a repeat dose of GnRHa at this critical hour (12 hr) could preserve the amplitude and the duration of gonadotropin surge, thus optimizing the oocyte maturity (16). They obtained a significantly higher yield of MII oocytes along with a statistically significant lesser number of MIs and GV oocytes in the repeat dose group. Our study findings do not support these findings. In our study, maturity rate, MI, MII, and GV oocytes were not statistically different between the two study groups, which question the proposed mechanism in repeat dose protocol. Moreover, the LH levels measured at 12 hr post-trigger predicted oocyte maturity in Deepika and colleagues study (16). But this relationship was not approved in our analysis. So, we can say that LH level post-trigger cannot necessarily predict the oocyte maturity and so the second dose does not improve the outcome of oocyte maturity.

EFS is a complication of IVF. It's thwarting for participants and leads to cycle cancelation. The etiopathophysiology of EFS is still unknown. It is reported as a complication in single-dose GnRHa trigger. Pituitary gland is the location of action for GnRHa and dysfunctions of the hypothalamic-pituitary-ovarian axis might not make optimal flare leading to deficient final oocyte maturation and EFS (17). We proposed that a second dose of GnRH agonist after 12 hr. since the first dose could provide duration of surge and amplitude and as a result of that may improve oocyte maturity after the GnRHa trigger. In our participants, no EFS was reported to support our hypothesis.

### Limitation

Our study had some limitations such as small sample size and missing reproductive outcomes. Overall, although one previous study with a proof-of-concept RCT suggested that a repeat dose of GnRHa 12 hr following the first dose in PCOS undergoing IVF delivers a better cycle outcome in terms of maturity of oocytes, higher number of blastocysts, and clinical pregnancy improvement, our RCT study revealed no difference between the repeat-dose and single-dose GnRHa trigger. Therefore, more RCT studies with large sample size and long follow-up is needed to evaluate the repeat dose effect more precisely.

## 5. Conclusion

Although, the second dose of GnRH agonist after 12 hr since the first dose could provide duration of LH surge and amplitude and as a result no empty follicular syndrome was seen, the maturity rate, MI, MII, and GV oocytes were not different between the two study groups.

##  Conflict of Interest

The authors declare that there is no conflict of interest.
