# Correction to “Does the repeat dose of gonadotropin-releasing hormone agonist trigger in polycystic ovarian syndrome improve in vitro fertilization cycles outcome? A clinical trial study” [Int J Reprod BioMed 2020; 18: 485-490]

**DOI:** 10.18502/ijrm.v20i7.11562

**Published:** 2022-08-08

**Authors:** Abbas Aflatoonian, Fatemeh Haghighi, Masrooreh Hoseini, Saeid Haghdani

The authors of the article entitled “Does the repeat dose of gonadotropin-releasing hormone agonist trigger in polycystic ovarian syndrome improve in vitro fertilization cycles outcome? A clinical trial study" requested some corrections in their article due to re-analysis of their data. The authors reviewed the data and confirmed that critical but inadvertent statistical analysis errors occurred during the research. As the authors explain in their letter to the editor, the errors are listed as:

• The recruitment end date has been changed from July 2018 to December 2018.
• The word `double-blind' was removed from the abstract and text.
• The word `severe' was added in front of `endometriosis' in the exclusion criteria.
• The Chi-square test has been added in the subtitles of table I and table II.
• As shown below, some numbers in table I have been changed (the mean and standard deviation of estradiol for group B and of LH1 for group B; the standard deviation of estradiol for group A, progesterone for group A, LH1 for group A and progesterone for group B; and the p-values for duration of infertility, estradiol and progesterone); the OHSS data from the original table I have been moved to table II; and the statistical tests have been revised.
• As shown below, some numbers in table II have been changed (the standard deviation of MI, MII, GV and LH4 for group A and of LH4 for group B; the mean and standard deviation of MI, MII and GV for group B; and the p-values for maturity rate, IVF, ICSI, 2PN, embryo, LH2 and LH3); the OHSS data from the original table I have been moved to table II; the statistical tests have been revised; and median values have been added.

**Original Table 1:** Comparison of patients characteristics between group A and group B


**Variables **	**Group A (n = 49) **	**Group B (n = 41)**	**p-value**
**Age**	29.30 ± 4.15	28.95 ± 5.23	0.72 ${}^{*}$
**BMI**	22.29 ± 4.03	22.27 ± 4.14	0.98 ${}^{*}$
**Duration of infertility**	6.32 ± 3.11	5.42 ± 3.18	1.8 ${}^{*}$
**Primary infertility N (%)**	43 (87.8)	35 (85.4)	0.74 ${}^{*}$
**Secondary infertility**	6 (12.2)	6 (14.6)	
**OHSS**	34 (69.4)	27 (65.9)	0.72 ${}^{*}$
**AMH**	8.68 ± 3.92	7.48 ± 3.81	0.14 ${}^{*}$
**Estradiol**	6543.22 ± 588.76	4912.16 ± 333.41	0.04 ${}^{**}$
**Progesterone**	1.17 ± 0.15	1.11 ± 0.17	0.86 ${}^{**}$
**LH1**	2.08 ± 0.22	3.02 ± 0.36	0.03 ${}^{**}$
Data presented as Mean ± SD BMI: Body mass index**;** OHSS: Ovarian hyper stimulation syndrome; AMH: Anti Mulerian hormone; LH: Luteinizing hormone; Independent sample *t* test*, Mann-Whitney U-test**			

**New Table 1:** Comparison of patients characteristics between group A and group B


**Variables **	**Group A (n = 49) **	**Group B (n = 41)**	**p-value**
**Age**	29.30 ± 4.15	28.95 ± 5.23	0.72 ${}^{*}$
**BMI**	22.29 ± 4.03	22.27 ± 4.14	0.98 ${}^{*}$
**Duration of infertility**	6.32 ± 3.11	5.42 ± 3.18	0.18 ${}^{**}$
**Primary infertility N (%)**	43 (87.8)	35 (85.4)	0.74 ${}^{***}$
**Secondary infertility**	6 (12.2)	6 (14.6)	
**AMH**	8.68 ± 3.92	7.48 ± 3.81	0.14 ${}^{**}$
**Estradiol**	6543.22 ± 4121.38	4941.91 ± 2090.84	0.02 ${}^{**}$
**Progesterone**	1.17 ± 1.08	1.11 ± 1.06	0.77 ${}^{**}$
**LH1**	2.08 ± 1.59	2.98 ± 2.27	0.03 ${}^{**}$
Data presented as Mean ± SD, BMI: Body mass index**;** OHSS: Ovarian hyper stimulation syndrome; AMH: Anti Mulerian hormone; LH: Luteinizing hormone; Independent sample *t* test*, Mann-Whitney U-test**, Chi-square test***			

**Original Table 2:** Comparison of outcomes between group A and group B


**Variables **	**Group A (n = 49)**	**Group B (n = 41)**	**p-value**
**Maturity rate**	0.80 ± 0.21	0.79 ± 0.18	0.89 ${}^{*}$
**Oocyte number**			
**MI**	1.28 ± 0.28	1.62 ± 0.54	0.38 ${}^{**}$
**MII**	19.83 ± 1.17	15.02 ± 1.10	0.06 ${}^{**}$
**GV**	3.02 ± 0.55	1.72 ± 0.27	0.38 ${}^{**}$
**ART method**			
**IVF**	6.89 ± 9.37	5.00 ± 6.66	0.28 ${}^{*}$
**ICSI**	12.85 ± 7.20	10.41 ± 5.63	0.08 ${}^{*}$
**2PN**	10.93 ± 7.29	9.46 ± 6.60	0.32 ${}^{*}$
**Embryo**	9.95 ± 7.23	8.51 ± 5.48	0.29 ${}^{*}$
**LH2**	50.29 ± 29.19	50.60 ± 35.21	0.96 ${}^{*}$
**LH3**	15.75 ± 9.77	15.60 ± 11.25	0.94 ${}^{*}$
**LH4**	4.71 ± 0.43	6.10 ± 1.11	0.66 ${}^{**}$
Data presented as Mean ± SD, MI: Meiosis I, MII: Meiosis II, GV: Germinal vesicle, IVF: In vitro fertilization, ICSI: Intra cytoplasmic sperm injection, 2PN 2 pronuclear, LH: Luteinizing hormone; Independent sample *t* test*, Mann-Whitney U-test**			

**New Table 2:** Comparison of outcomes between group A and group B


**Variables **	**Group A (n = 49)**	**Group B (n = 41)**	**p-value**
**OHSS **	34 (69.4)	27 (65.9)	0.72 ${}^{***}$
**Maturity rate**	0.80 ± 0.21, MD = 00.86	0.79 ± 0.18, MD = 00.84	0.37 ${}^{**}$
**Oocyte number**			
**MI**	1.28 ± 2.00, MD = 00.00	1.60 ± 3.37, MD = 1.00	0.38 ${}^{**}$
**MII**	19.83 ± 12.2, MD = 16.00	14.90 ± 6.97, MD = 14.00	0.06 ${}^{**}$
**GV**	3.02 ± 3.88, MD = 2.00	1.80 ± 1. 77, MD = 2.00	0.38 ${}^{**}$
**ART method**			
**IVF**	6.89 ± 9.37, MD = 0.00	5.00 ± 6.66, MD = 0.00	0.58 ${}^{**}$
**ICSI**	12.85 ± 7.20, MD = 11.00	10.41 ± 5.63, MD = 10.00	0.13 ${}^{**}$
**2PN**	10.93 ± 7.29, MD = 9.00	9.46 ± 6.60, MD = 9.00	0.43 ${}^{**}$
**Embryo**	9.95 ± 7.23, MD = 8.00	8.51 ± 5.48, MD = 8.00	0.60 ${}^{**}$
**LH2**	50.29 ± 29.19, MD = 40.80	50.60 ± 35.21, MD = 41.15	0.91 ${}^{**}$
**LH3**	15.75 ± 9.77, MD = 14.20	15.60 ± 11.25, MD = 11.35	0.51 ${}^{**}$
**LH4**	4.71 ± 3.06, MD = 3.70	6.10 ± 7.02, MD = 3.85	0.66 ${}^{**}$
Data presented as Mean ± SD, MI: Meiosis I; MII: Meiosis II; GV: Germinal vesicle; IVF: In vitro fertilization; ICSI: Intra cytoplasmic sperm injection; 2PN 2 pronuclear; LH: Luteinizing hormone, Chi-square test***, Mann-Whitney U-test**, MD: Median			

One of the readers of the article informed the corresponding author that there seemed to be a number of errors in presenting the data, so the authors re-analyzed the data and due to the selection of an inappropriate statistical test, some numbers were incorrect. The corrected article has been provided with corrections to the paper and relevant tables. The authors have confirmed that there are no other errors. The corrected article has been reviewed by our editorial team, and we have confirmed that the overall conclusion has not been changed as stated in the updated article available at: https://doi.org/10.18502/ijrm.v13i7.7363 (updated on July 28, 2022).

